# The Role of HbA1c in the Follow-Up and Control of Diabetes Mellitus

**DOI:** 10.7759/cureus.78357

**Published:** 2025-02-01

**Authors:** Soliman S Ghanem, Eman M Abdulkreem, Muath S Alfurayh, Amani A Ahmed, Hind A Rikabi

**Affiliations:** 1 Basic Sciences, College of Pharmacy, Rafha, SAU; 2 Natural Products and Alternative Medicine, College of Pharmacy, Rafha, SAU; 3 Pharmacology, Al-dawaa Pharmacy, Rafha, SAU; 4 Pharmacology, College of Pharmacy, Rafha, SAU

**Keywords:** control, diabetes mellitus, follow-up, glycosylated, lifestyle modifications

## Abstract

Introduction

HbA1c is a biomarker that plays an essential role in the diagnosis and follow-up of diabetic patients. The sensitivity and specificity of the biomarker may be affected by common comorbidities seen in individuals with diabetes mellitus, such as kidney affection, cardiac affection, iron deficiency anemia, hemolytic anemia, hemoglobinopathies, and drug consumption.

Aim

To assess the role of HbA1c in the follow-up and control of Diabetes Mellitus across different patient populations and clinical scenarios.

Methodology

A quantitative research design to investigate the impact of residence location (inside or outside the Saudi Arabia). Through an electronic questionnaire in English and translated into Arabic, it was published online using Google Forms (Google, Mountain View, California) and was divided into three sections.

Results

The number of participating patients was 412 diabetic patients; their average age was from 51 to 60 years, and most of them were male. The study found a high level of awareness among diabetics about the HbA1c test, their knowledge of its importance, and their ability to perform the analysis regularly and periodically every year. However, there is a lack of practice, as most participants only performed follow-up every more than a few months, and the value of the last HbA1c test ranged between seven and ten. The percentage of diabetic patients whose cumulative blood sugar was more than ten and who did not take any action after learning of their high percentage (35.7%).

Conclusion

Diabetes is one of the most common health problems that continue to spread worldwide. HbA1c testing may continue to be implemented as an essential part of the diagnostic and prognostic tool, resulting in better patient care and successful clinical outcomes. Testing for glycosylated hemoglobin levels should be done every three months. Patients and doctors should be cautioned not to rely exclusively on two-hour post-prandial blood glucose results, particularly in the case of insulin-dependent diabetes.

## Introduction

Diabetes mellitus (DM) is one of the most common health-challenging problems worldwide, with its prevalence steadily rising across all age groups and geographical regions. It's a chronic metabolic disorder characterized by increased blood glucose levels. It is also considered a chronic silent killer, and systemic disease as it affects the whole body organs. Effective treatment and regular follow-up are essential to prevent or reduce the complications of this common health problem. Glycemic control plays a central role in achieving these goals [1].

Diabetes mellitus encompasses a group of metabolic disorders characterized by hyperglycemia due to either relative or absolute deficiency of insulin hormone. Type 1 DM is characterized by autoimmune destruction of pancreatic beta cells, leading to absolute insulin deficiency, while type 2 DM involves insulin resistance and relative insulin deficiency [2].

The effect of uncontrolled diabetes is very frightening because it affects all body organs, especially vital organs such as the heart, kidneys, and brain. Effective management of DM requires a non-pharmacological approach that includes patient education, diet control, exercise, and general lifestyle modifications, in addition to pharmacotherapy and regular monitoring [3].

In fact, there are different biomarkers used to assess glycemic control. Hemoglobin A1c (HbA1c) has emerged as a key biomarker due to its ability to reflect average blood glucose levels over a long period (three months). HbA1c is formed by the non-enzymatic glycation of hemoglobin in red blood cells, and its levels are influenced by the prevailing glucose concentrations in the blood. It can be performed at any time of the day and does not require any special preparation, such as fasting in fasting plasma glucose levels or performing an oral glucose tolerance test (OGTT) and day-to-day variability in glucose [4]. HbA1c has now been recommended by an International Committee and by the Americans with Disabilities Act (ADA) to diagnose diabetes. It is also used as a screening test for persons at high risk of diabetes [5]. As such, HbA1c provides valuable insights into long-term glycemic control and is widely used in clinical practice to guide DM management [6]. 

Despite the known importance of HbA1c in DM management, there are several areas of interest, such as the relationship between the educational level and the awareness about the value and importance of HbA1c in regular follow-up and controlling of diabetes mellitus and the optimal target HbA1c levels for different patient populations and comorbidities remains a topic of debate, with varying guidelines and recommendations across international organizations. Understanding the impact of achieving and maintaining specific HbA1c targets on clinical outcomes is essential for controlling and regular follow-up of diabetic patients [7].

Additionally, there is an increase in awareness regarding the role of HbA1c variability in predicting complications and treatment response. The variability in HbA1c levels, independent of average HbA1c values, has been associated with increased cardiovascular risk and other adverse outcomes. The detection of the clinical effect of HbA1c variability can help us in changing our approach to DM management and risk evaluation [8].

Furthermore, advancements in HbA1c measurement techniques, including standardized assays and point-of-care devices, have improved the accuracy and accessibility of HbA1c testing. Evaluating the impact of these technological advancements on clinical decision-making and patient outcomes is vital for optimizing diabetes care delivery [9].

Research objectives

The objective of this research was to assess the role of HbA1c in the follow-up and control of diabetes mellitus across different patient populations and clinical scenarios, in addition to investigating the relationship between HbA1c levels with patients' demographics (such as age, level of education, marital status) and clinical outcomes, including diabetic complications and treatment response.

## Materials and methods

A quantitative cross-sectional study was conducted during a period of four months between March and June 2023. A total of 412 adults and elderly people, 284 (68.9%) male and 128 (31.6%) female, residing in the Northern Borders Region - Kingdom of Saudi Arabia, with regular follow-up visits (every three months, six months, one year, or every five years) on HbA1c levels among diabetic patients.

Sample selection was made according to the inclusion criteria: diabetic patients residing in the Northern Borders Region - KSA; aged 18 years old or more; some of them diagnosed as diabetic patients for less than 10 years and others for more than 10 years duration. Exclusion criteria included patients with comorbidities affecting HbA1c levels (e.g., hemoglobinopathies) and patients with incomplete medical records or missing HbA1c test results.

Data was collected through an electronic questionnaire in English and translated into Arabic, specially designed for research purposes. It was published online using Google Forms (Google, Mountain View, California) and divided into three sections: a demographic information section, therapeutic properties of diabetes, and a section to determine the level of knowledge of diabetics with the cumulative blood sugar test.

Consent was obtained from each participant before data collection.

## Results

Demographic characteristics of the study participants

A total of 412 adults and elderly people residing in the Kingdom of Saudi Arabia participated in the survey. Their characteristics are shown in Table 1. Most of the participants were male 284 (68.9%), female 128 (31.6 %) aged 51 to 60 years (48.1%), followed by those under the age of 50 (37.9%). Their level of education varied, with 44.7% having a Tertiary School degree and 41.3% of them having university degrees. Most of them reside within the Northern Borders Region (NBR), Kingdom of Saudi Arabia (98.5%), and only 1.5% outside it. Most of the participants were married (300, 72.8%) & about 112 non-married (27.2). Table 2 shows the action taken by patients whose percentage exceeded 10 degrees, which shows that 21.4% took insulin, 21.4% followed a diet, and 14.3% exercised, while 35.7% did not take any action after knowing their high percentage, and this is due to the necessity of raising awareness for diabetics about the necessity of adhering to the treatment prescribed to them according to their health condition.

**Table 1:** Demographic characteristics of the study participants (n=412)

Characteristic		Number	Percentage
Gender	Female	128	31.6%‎
	Male	284	68.9‎%‎‎‎
Age	<50	156	37.9‎%‎
	From 51 to 60	198	48.1‎%‎
	>60	58	14.1‎%‎
Education Level	Primary school	16	3.9%
	Secondary school	22	5.3%
	Tertiary school	184	44.7‎%‎
	University or college	170	41.3‎%‎
	Master/PhD	20	4.9%
Marital status	Married	300	72.8
	Not married	112	27.2
Location of residency	NBR (KSA)	406	98.5‎%‎
	Outside	6	1.5%

**Table 2:** Participants dealt with the high percentage more than 10 (n=28)

Question	Response	Number	Percentage
What did the result of your cumulative blood sugar test the last time you took it?	Nothing	10	35.7‎%‎
	Insulin injection	6	21.4‎%‎
	Sports	4	14.3‎%‎
	Dietary pattern	6	21.4‎%‎
	All of them	2	7.1‎%‎

Diabetes treatment characteristics of the study participants

The largest percentage had been suffering from diabetes for more than 10 years, and 40.3% of the participants had been suffering from diabetes for less than 10 years; 55.8% take tablets (oral hypoglycemic drugs) only for treatment, and 12.6% use tablets and insulin together for treatment, while 8.3% of them use insulin only, and 12.6% do not follow any treatment for diabetes. 84.5% of the participants had anti-diabetic medication adherence. More than half of the participants did not exercise (56.8%), whereas 43.2% were committed to exercising (Table 3).

**Table 3:** Participants characteristics about diabetes treatment (n=412)

Characteristic		Number	Percentage
Duration of diabetes (in years)	≤10	166	40.3‎%
	>10	246	59.7‎%‎
Type of treatment	No treatment	52	12.6‎%‎
	Diet only	44	10.7‎%‎
	Oral hypoglycemic tablets	230	55.8‎%‎
	Insulin only	34	8.3%
	Tablets and insulin	52	12.6‎%‎
Anti-diabetic medications adherence	Yes	348	84.5‎%‎
	No	64	15.5‎%‎
Do you practice sports?	Yes	178	43.2‎%‎
	No	234	56.8‎%‎
Number of follow-up visits	Less than 3 months	114	32.5‎%‎
	More than 3 months	276	67.5‎%‎
Follow-up visits place	Home	44	10.9‎%‎
	Hospital	358	89.1‎%‎
Co-morbidities	No	162	39.3‎%‎
	Hypertension	178	43.2‎%‎
	Hypercholesterolaemia	44	10.7
	Others	28	6.8
Complications	No complications	346	84‎%‎
	Nephropathy	8	1.9%‎
	Retinopathy	24	5.8%‎
	Neuropathy	20	4.9%‎
	Others	14	3.4

Most of the participants (67.5%) perform the follow-up every more than a few months; 89.1% perform the follow-up in the hospital, while only 10.9% perform the follow-up from home.

Table 4 shows patients' information and knowledge of the ultra HbA1c test quantitatively, which showed that most of the participants (93.2%) knew about the HbA1c test, and 58.3% of them knew its importance. Most of the participants (62.3%) were taking the HbA1c test on a regular basis every year, and 17.6% of them had done it several times, while 9.3% had not done it at all. 88.7% explained that the HbA1c test helped them control their sugar levels. The last time the participants took the cumulative blood sugar test, its value was between 7 and 10 for 57.4% of them and less than 7 for 37.6% of the participants, while it was higher than 10 for only 5% of the participants.

**Table 4:** Participants knowledge about HbA1c test (n=412)

Characteristic	HbA1c	Number	Percentage
Have you heard of the HbA1c test?	Yes	384	93.2‎%‎
	No	28	6.8%
Do you know the importance of the HbA1c test?	Yes	240	58.3‎%‎
	No	172	41.7‎%‎
How many times have you done the HbA1c test?	No one	38	9.3‎%‎
	Once only	44	10.8‎%‎
	2-3 times	72	17.6‎%‎
	On a regular basis every year	254	62.3‎%‎
Did you feel that this test helped you control your HbA1c levels?	Yes	362	88.7‎%‎
	No	46	11.3‎%‎
What was the result of your cumulative HbA1c test the last time you took it?	<7	152	37.6‎%‎
	7-10	224	57.4‎%‎
	>10	28	5%
Married (n=300)	<7	150	50%
	7-10	100	30%
	>10	50	20%
Not married (n=112)	<7	50	44.6%
	7-10	44	39.3%
	>10	18	16%

Table 5 shows that there is a clear relationship between the education level and controlling of the blood sugar level; there is a marked decrease in the level of HbA1c with highly educated participants.

**Table 5:** Participants education level and HbA1c level

Education level	No	%	HbA1c level
Primary school	16	3.9%	>10
Secondary school	22	5.3%	>10
Tertiary school	184	44.7‎%‎	(n=100) >10
			(n=84) 7-10
University or college	170	41.3‎%‎	<7
Master/PhD	20	4.9%	<7

Table 2 shows the action taken by patients whose percentage exceeded 10 degrees, which shows that 21.4% took insulin, 21.4% followed a diet, and 14.3% exercised, while 35.7% did not take any action after knowing their high percentage, and this is due to the necessity of raising awareness for diabetics about the necessity of adhering to the treatment prescribed to them according to their health condition.

## Discussion

In our study to evaluate the role of HbA1c analysis in monitoring and controlling the treatment of diabetes in the Kingdom of Saudi Arabia, the number of participating patients was 412 diabetic patients; their age range was from 51 to 60 years, and most of them were male.

The current study found a high level of awareness among diabetics about the HbA1c test; their knowledge about its importance is highly good, especially among the highly educated participants (Table 4), and their ability to perform the analysis regularly and periodically every year.

Similarly, another study agrees with our findings and noted that the awareness of the glycosylated hemoglobin (HbA1c) test among diabetic patients, especially those with type 2 diabetes mellitus, was higher with increasing education levels and those with higher income [10].

Most participants were conducting follow-ups every few months, and the value of the last HbA1c test was between seven and ten. Therefore, regular follow-up visits must be maintained because it is important to manage HbA1c and TC levels and reduce the risk of diabetes complications in the future.

In the current study, another finding explains that the married participants (n=300) had a good awareness level about the importance of HbA1c in controlling and following up on their glycemic level, as it appears clear from their level of HbA1c (Table 3). This point is explained as both partners helped each other in many points, such as healthy diet and exercise programs, medical adherence, and regular follow-up.

Another study disagrees with the current study, explaining that married participants had higher risks of unsuccessful glycemic control than single respondents [11].

Our study also explains that the diabetic patients whose cumulative blood sugar test was more than ten and who did not take any action after knowing their high percentage mostly had a low educational level, were housewives, and lived in villages (rural areas). In this condition, we need to arrange for repeated campaigns to increase the level of awareness about the necessity of adhering to treatment and the necessity of following a diet and exercising regularly for diabetics.

Another similar study explains that the prevalence of poor control was higher among females (p=0.003), participants with a lower level of education (p=0.001), those living in rural areas (p<0.001), and unemployed people or housewives (p=0.011). Further, the prevalence of poor glycaemic control was higher among smokeless tobacco consumers (p=0.008) and unhealthy diners (p=0.008) [12].

A higher prevalence of poor glycaemic control was present among people with more than five years' duration of diabetes (p = 0.005), insulin users (p<0.001), those having irregular follow-up check-ups (p<0.001), patients with a history of coronary artery diseases (CAD) (p=0.001), and those with cognitive impairments (p<0.001) [13].

## Conclusions

Diabetes is one of the most common health problems that continue to spread worldwide; HbA1c testing may continue to be implemented as an essential part of the diagnostic and prognostic tool but must be associated with extensive awareness health education programs through media as this study shows there is a high level of awareness but of little value, as only 18 participants out of nearly 406 participants dealt with the high percentage of HbA1c and take action in the form of more restricted diet control, exercise program. More studies should also be implemented in this area to explain the relationship between the level of HbA1c and other variables such as comorbidities and culture.
